# One-step synthesis of polyethyleneimine-coated magnetite nanoparticles and their structural, magnetic and power absorption study

**DOI:** 10.1039/d0ra08872b

**Published:** 2020-11-17

**Authors:** Lizbet León Félix, Marco Antonio Rodriguez Martínez, David Gregorio Pacheco Salazar, José Antonio Huamani Coaquira

**Affiliations:** Laboratorio de Películas Delgadas, Escuela Profesional de Física, Universidad Nacional de San Agustín de Arequipa Av. Independencia s/n Arequipa Peru lleonf@unsa.edu.pe; Laboratory of Magnetic Characterization, Instituto de Física, Universidade de Brasília DF 70910-900 Brasília Brazil

## Abstract

Magnetic nanoparticles (NPs) are especially interesting for several biomedical applications due to their chemical surface, especially for targeted cancer imaging and therapeutics. In order to realize these applications, it is important to know their magnetic properties among other complementary properties that help to improve the understanding of the synthesis process. In this work, we report the magnetic properties of polyethyleneimine-coated magnetite (PEI-Fe_3_O_4_) NPs synthesized by a one-step method *via* the co-precipitation method and using PEI as a stabilizer. Transmission electron microscopy (TEM) images revealed agglomerated magnetic nanoparticles with an average size of ∼10 nm; meanwhile, the X-ray diffraction (DRX) analysis confirmed a pure magnetite phase. The study of magnetic properties shows a superparamagnetic system with coexistence of non-interacting single NPs with a low blocking temperature (∼35 K) and interacting NPs in the aggregates with a higher blocking temperature (>150 K), in which the interparticle interactions of magnetic cores dominate over surface spin disorder. The interaction between the surface spin-disorder layer and NP core was found to be weak, related to a weak exchange bias effect. A maximum specific loss power (SLP) value of 70 W g^−1^ was obtained (*f* = 571 kHz and *H* = 23.87 kA m^−1^) indicating that the magnetic response plays a crucial role in determining the heating efficiency for future applications.

## Abstract 2

Magnetic nanoparticles (NPs) are especially interesting for several biomedical applications due to their chemical surface, especially for targeted cancer imaging and therapeutics.

## Graphical abstract

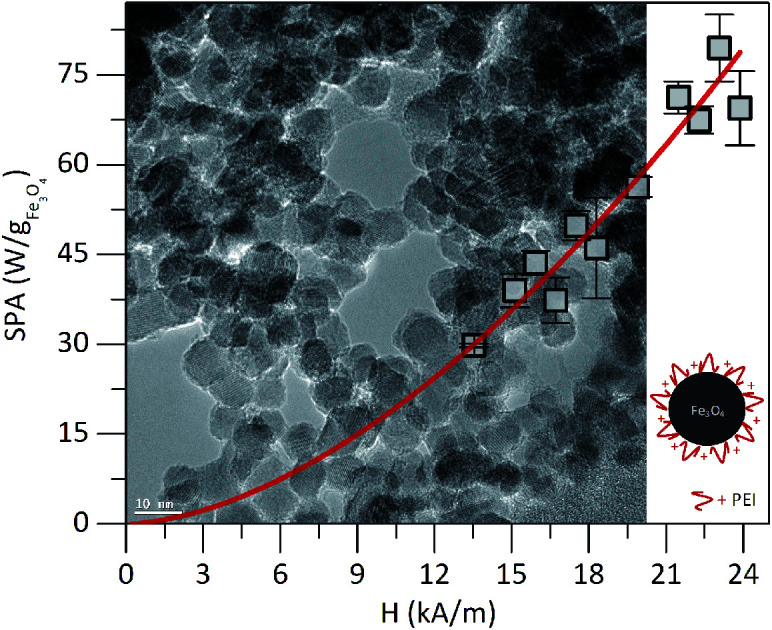

## 1. Introduction

Iron oxide magnetic NPs with an appropriate biocompatible coating to improve colloidal stability^1^ and a good biocompatibility or low cytotoxicity are increasingly used in many biomedical applications including protein separation,^2^ drug and gene delivery,^3^ magnetic resonance imaging (MRI)^4^ and magnetic hyperthermia therapy.^5,6^

Magnetic hyperthermia is an alternative to therapeutic treatments which uses hysteresis cycle of magnetic nanoparticles (MNPs) to locally increases cancer cells temperature to values of 43–45 °C inducing their apoptosis or death.^7^ The heating capacity of MNPs with colloidal stability under an alternating magnetic field (AMF) is quantified by the specific loss power (SLP).^8^ In order to improve the heating process, it is important to know the influence of the different parameters such as size and shape of NPs, solvent, colloidal stability, NPs biocompatibility, and intrinsic magnetic properties. Those parameters can lead to a more complex behavior of the system that could affect the heating efficiency of the NPs.

Also, it is important to consider the influence of the surface, interface effects such as surface spin disorder, defects, breaking of exchange bonds, changes in the surface atom coordination number,^9^ and also the distance among MNPs assemblies that leads to interparticle interactions^10^ that can dominate over single domain nanoparticles response and affect the heating efficiency.

Some nanoparticulated features such as the surface spin disorder and magnetic frustrations, related to the presence of structurally disordered grain boundaries, can provide a dominant contribution to the effective anisotropy and lead to a surface spin-glass like state at low temperatures.^11^ It is known that the occurrence of a spin-glass state is related to a magnetic disorder, randomness (exchange, anisotropy, field), and frustrations,^12^ where the competing interactions (ferromagnetic and antiferromagnetic) show equivalent strengths.

In addition, in a system where interparticle interactions are non-negligible, the system eventually shows collective behavior, which overcomes the anisotropy properties of individual particles, and leads to the increase of the blocking temperature (*T*_B_). When the interparticle interactions are strong enough in a nanoparticle ensemble,^13^ also can lead to the spin-glass behavior besides the increment of the *T*_B_.^14^

In particle systems, collective effects related to different kinds of magnetic interactions play an important role such as the long-range dipole–dipole interactions that are the predominant mechanism; meanwhile, the short-range exchange interactions play a leading role in nanoparticle assemblies, where the electrons at the surface of the particles are in close contact^12^ and both mechanisms may exist simultaneously. De Toro *et al.* reported that despite the close contact of the maghemite particles (with nonmagnetic shell thickness < 3 nm) the superexchange interactions play a minor role in establishing the collective, and superspin-glass state of the NPs below a critical temperature in comparison to dipolar interactions.^15^ The origin of the superspin-glass behavior is strongly related to the interplay of intra- and interparticle interactions effects, where the role of dipolar interactions is very important for establishing the superspin-glass phase.^14^

Magnetic NPs systems can exhibit the so-called exchange bias (EB) effect, which is related to the exchange coupling between core and surface spins at the interface and the interparticle exchange coupling. A study of NPs with Fe/Fe oxide core (10 nm)/shell (3.5 nm) structure shows a superparamagnetic behavior and the structural disorder in particles outer shell could lead to a larger number of uncompensated spins at the interface of the core–shell structure, which, in turn, causes a high magnetocrystalline anisotropy and an enhanced EB effect (22 kA m^−1^ at 5 K and using a field of 1591.5 kA m^−1^ to cool the system).^16^ On the other hand, it has been reported an EB effect for a core–shell structure of Fe_3_O_4_/γ-Fe_2_O_3_ NPs of 12 nm size with an EB field of 11.14 kA m^−1^ at 10 K and after field cooling with 37.8 kA m^−1^ (ref. 17) the authors reported that there is an intrinsic single-particle property, as the large magnetic anisotropy of the γ-Fe_2_O_3_ shell with a spin glass-like behavior or a possibly disordered magnetic state no related to the interparticle interaction.^18^ Meanwhile, the observed EB effect in ultrasmall ∼2 nm MnFe_2_O_4_ NPs has been assigned to the exchange coupling between core and surface spins at the interface and the interparticle exchange coupling.^16^ In magnetite NPs of 40 nm a spin-glass like behavior with a freezing temperature of ∼35 K was determined and could be observed in both, the in-phase and the out-phase magnetic susceptibility curves.^19^ These characteristics can be tuned during the synthesis process.

So far, various experimental methods have been employed to produce PEI coated magnetic NPs such as solvothermal, hydrothermal, and co-precipitation methods. They have been applied for the synthesis at high temperatures,^20^ during long periods of synthesis^6^ using several reaction steps,^21,22^ such as first core synthesis, and after coated with PEI.^23–27^ PEI molecule is considered a good candidate for the functionalization of other molecules, the biocompatibility and stability of MNPs, that are important in the fields of biomedicine.^28^

In order to improve and simplify the synthesis for the formation of PEI-coated MNPs, we propose an effective procedure that combines co-precipitation steps in a one-step procedure in an aqueous medium. The design of PEI-MNPs has a huge importance in the heat generation *via* electromagnetic energy conversion.

In this work, we report a simple route for an efficient and facile one-step PEI-Fe_3_O_4_ NPs synthesis, using the co-precipitation method. We obtained NPs of ∼9.4 nm in size, and the study of their magnetic properties and the power absorption response are presented and discussed. The phase, crystal structure, and magnetic properties were characterized by X-ray diffraction, transmission electron microscopy, dc, and ac magnetic measurement and power absorption for future applications in magnetic hyperthermia.

## 2. Experimental section

### 2.1 Chemicals

Iron(iii) chloride hexahydrate (FeCl_3_·6H_2_O), iron(ii) chloride tetrahydrate (FeCl_2_·4H_2_O), sodium hydroxide (NaOH) and polyethylenimine branched (PEI) with a molecular weight of ∼25 000 were purchased from Sigma Aldrich.

### 2.2 Synthesis of polyethyleneimine coated magnetite nanoparticles

The synthesis protocol used for the samples was based on a co-precipitation method. At the beginning of the synthesis, 8 mol L^−1^ NaOH solution (water) was heated up to 90 °C by vigorous constant stirring and bubbled with N_2_. After 10 min, the mixture of 1 mol L^−1^ FeCl_2_ and 2 mol L^−1^ FeCl_3_ with 2.1 g PEI (25 kDa) was dissolved in distilled water and was added in a basic solution while constantly stirring. Then the system was maintained at 90 °C for 2 h under N_2_. When the precipitation was completed, the suspension with black precipitate was removed from the heating source and cooled at room temperature with an ice bath then washed with water several times in order to isolate the supernatants by magnetic decantation, and re-dispersed in water until getting pH 7, the particle presented a good water dispersibility and stability. The final product was dried with N_2_ flux.

### 2.3 Characterization

The morphology and structure of the resulting NPs were analyzed by a high resolution transmission electron microscopy (HRTEM), using an FEI Tecnai F30 microscope operated at an acceleration voltage of 300 kV. Lattice fringes were measured from the fast-Fourier transform of HRTEM images analyses, using Gatan Digital Micrograph software. The mean particle size, and its polydispersion index, *σ*, were obtained by calculating the average number manually measuring the equivalent diameter of *N* > 500 particles from TEM micrographs. XRD patterns were obtained using a Rigaku Miniflex 600 diffractometer operating at 30 mA and 40 kV from 20 to 80° (2*θ* value) using Cu K-α radiation (0.15418 nm). The samples were prepared placing a concentrated NPs suspension drop on a zero-diffraction silicon wafer. The Rietveld method analysis was used to confirm the structural analysis of NPs. The lattice parameters were determined using the GSAS (General Structure Analysis System) refinement. The magnetic properties of the sample were measured by ac and dc magnetic susceptibility measurements, performed on a Superconducting Quantum Interference Device (SQUID) magnetometer, model MPMS3 (Quantum Design). The magnetization curves were recorded using a maximum applied field of 5570.4 kA m^−1^ at temperatures of 5 and 300 K. For zero-field-cooled (ZFC) and field-cooled (FC) curves were measured at temperatures between 2 to 300 K, with a cooling field *H*_FC_ = 2.39 kA m^−1^ (30 Oe). For ac magnetic susceptibility measurements, the data was obtained in a temperature range of 2 to 300 K, and frequencies ranging from 0.2 to 1 kHz and under an excitation field *H*_ac_ = 400 A m^−1^. The Specific Loss Power (SLP) of the SLP NPs was measured under AMFs as a function of the field amplitude (13.53 ≤ *H*_0_ ≤ 23.87 kA m^−1^) and a fixed frequency of *f* = 571 kHz. At 478 nm, the wavelength was used to measure the absorbance of NPs and for determining the iron concentration.

## 3. Results and discussion

### 3.1 Structural and morphological characterization

**Fig. 1:**
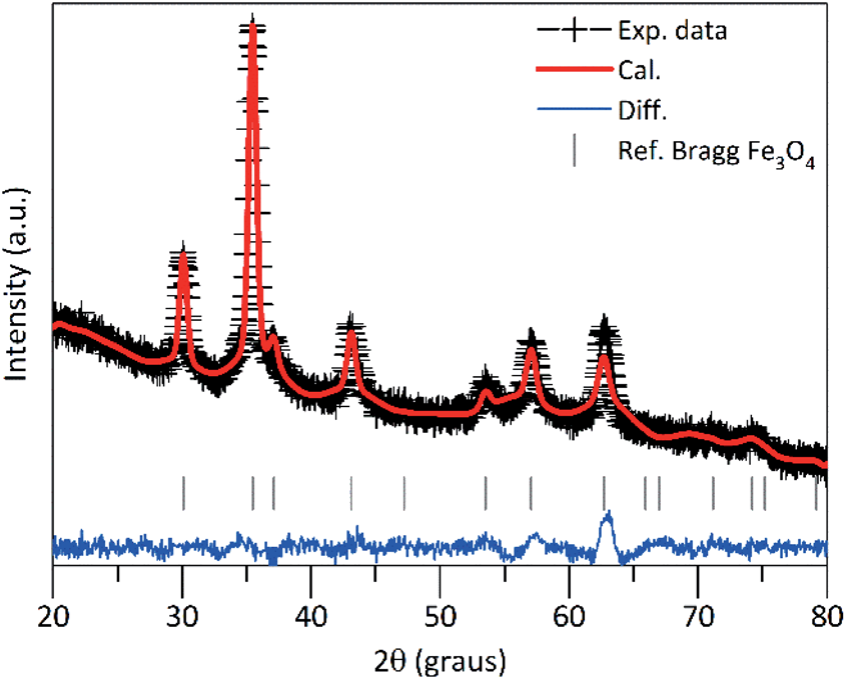
X-ray diffraction pattern of the PEI-Fe_3_O_4_ nanoparticles. The solid red line represents the calculated pattern and the experimental data are represented by the cross symbol; the blue line at the bottom of the plot represents the difference between the experimental and calculated data. The inserted vertical lines indicate the Bragg planes.

In Fig. 1 is shown the ray-X diffraction data analysis of the PEI-FE_3_O_4_ NPs. The XRD pattern was refined using the Rietveld refinement method, which indicates that all diffraction peaks correspond to the cubic spinel structure of magnetite (space group: *Fd*3̄*m*). The lattice parameter calculated from the XRD pattern is ≈8.37 Å, which is very close to the standard lattice parameter of bulk magnetite (8.39 Å). Additionally, the full width at half-maximum of the peaks was used to estimate the crystallite size (*D*_XRD_) from the Scherrer equation. From this analysis, the calculated value was *D*_XRD_ ≈ 10.1 nm.

**Fig. 2:**
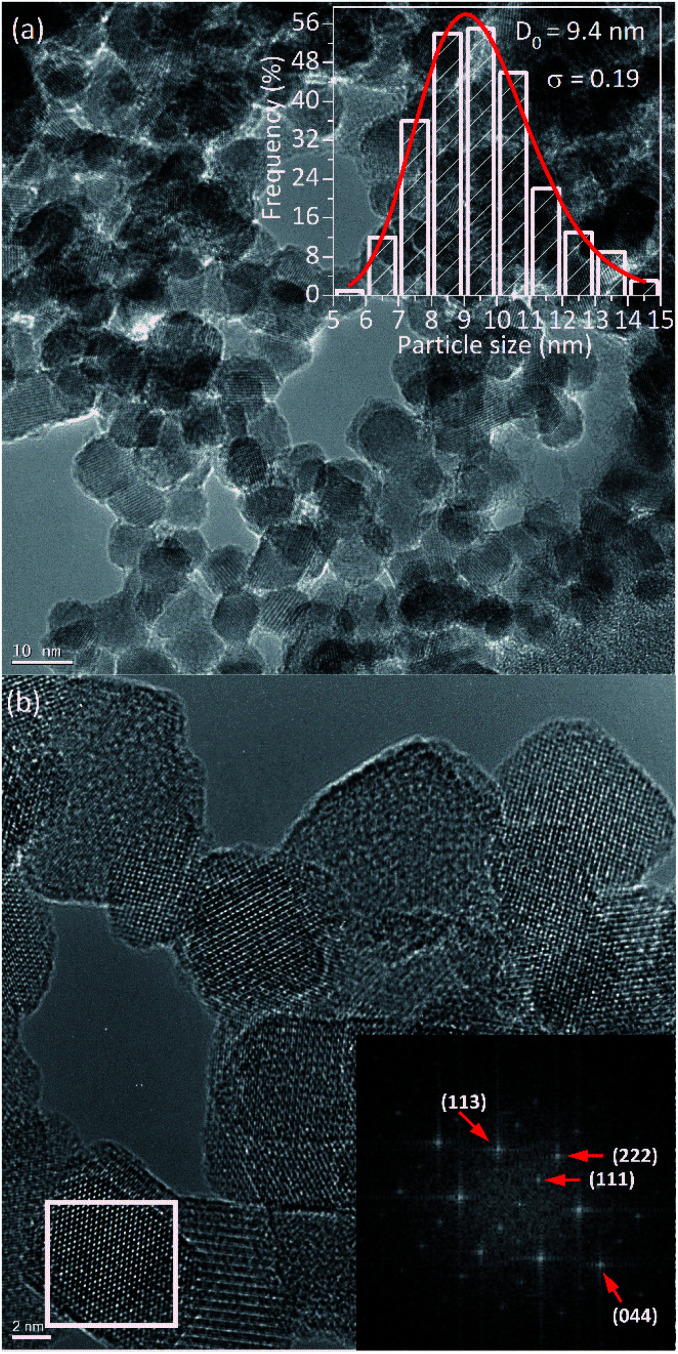
(a) TEM image of the PEI-Fe_3_O_4_ NPs. In the inset, the histogram of particle size distribution with lognormal functions is shown. (b) The high-resolution TEM image of the NPs with the corresponding Fourier transform of the selected region is shown at the lower part.

Fig. 2 shows the TEM images of PEI-coated Fe_3_O_4_ NPs, which were used to determine the structure, morphology, and size distribution. After counting *N* = 518 sizes, the histogram built using the Sturges criterion could be fitted with a log-normal distribution, yielding an average size of 〈*d*〉 = 9.6 nm and a polydispersion index *σ* = 0.19 nm (see Fig. 2a). As observed in the images, the NPs show mainly spherical and octahedral shapes, and also agglomeration of particles is observed that could mimics a large particle. Especially, Fig. 2b displays the high-resolution TEM images with clear lattice fringe patterns, which confirm the high crystalline quality of the sample. The Fourier transform of the HR-TEM images show spots corresponding to the spacing distances of 4.85 Å, 2.48 Å, 2.59 Å and 1.55 Å, which are consistent with the interplanar distances of (111), (222), (113) and (044) lattices planes of magnetite, respectively.

### 3.2 Magnetic characterization

In order to characterize the magnetic properties in detail, the static and dynamic magnetic responses were investigated. We begin the analysis with the estimation of the *T*_B_ of non-interacting particles, using the average size obtained by TEM and the relation, *T*_B_ = *K*_eff_*V*/25*k*_B_, where *k*_B_ is the Boltzmann constant, considering the anisotropy constant (*K*_eff_) of magnetite bulk. A mean value of 〈*T*_B_〉 ≈ 22 K was obtained.

**Fig. 3:**
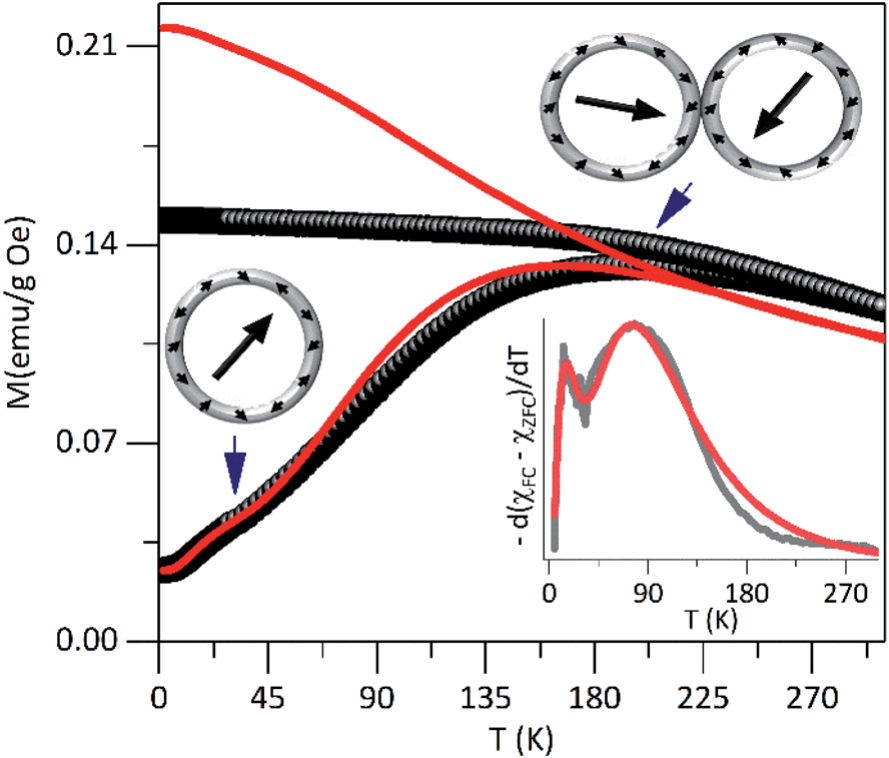
The dc magnetization curves in ZFC–FC modes for PEI-Fe_3_O_4_ NPs, carried out at 2.39 kA m^−1^. In the inset is shown the simulated data fit (red solid lines).

In Fig. 3 are shown zero-field-cooled (ZFC) and field-cooled (FC) magnetization curves of PEI-Fe_3_O_4_ NPs obtained with an applied field of 2.39 kA m^−1^. The ZFC curve shows, a broad maximum temperature at *T*_max_ ∼ 195 K and a shoulder in the low-temperature region at around 30 K. The high *T*_max_ value suggests the occurrence of strong dipolar interparticle interactions^29^ due to the NPs being very close to each other, forming particle aggregates as observed by TEM images, which favor the interparticle interactions. Moreover, the shoulder could be related to the blocking of non-interacting single particles. At temperatures below *T*_B_, the magnetic moments of the particles must show random orientation in a zero-field cooled condition and cannot rotate freely.^30^ Then, the system would exhibit a transition from a high-temperature superparamagnetic state to a low temperature blocked state.

The distribution of *T*_B_ can be obtained from ZFC–FC curves according to *f*(*T*) ≈ d(*M*_ZFC_ − *M*_FC_)/d*T*.^31,32^ As it is shown in the inset of Fig. 3, the experimental data shows bimodal features that suggest the occurrence of two *T*_B_ distributions, which can be modeled using the lognormal distribution. The fit provides two maxima at 〈*T*_1B_〉 ∼ 25 K and 〈*T*_2B_〉 ∼ 105 K. The 〈*T*_1B_〉 value is close to the one expected for non-interacting single particles with the determined size from TEM images, and 〈*T*_2B_〉 is not directly related to the particle size distribution but could be associated with the presence of interactions among close particles or particle agglomerations that promote the interparticle interactions. Those values obtained from the fit of the *f*(*T*) ≈ d(*M*_ZFC_ − *M*_FC_)/d*T vs. T* curve was used to simulate the ZFC and FC curves.

It is known that the effect of the particle size distribution gives rise to a distribution of *T*_B_, which can drive to the superposition of responses coming from the superparamagnetic state (first term in eqn (1) and (2)) and in the blocked state (second term in eqn (1) and (2)).^33,34^ Then, the ZFC (eqn (1)) and FC (eqn (2)) susceptibility can be given by:1
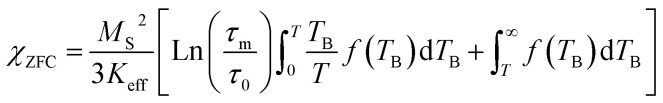
2
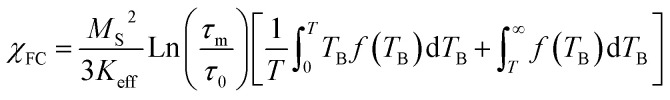
where *M*_S_ is the saturation magnetization, *K*_eff_ is the effective anisotropy constant, *f*(*T*_B_) is the distribution function of *T*_B_ and *τ*_m_ is the measuring time. In Fig. 3 are shown the simulated curves obtained using eqn (1) and (2).

These results confirm the presence of magnetic interactions among particles^35^ that displaces *T*_B_ to high temperatures. The magnetic interactions such as exchange interaction^36^ might be negligible due to particle separation, by the presence of PEI on the particle surface. However, the dipolar interparticle interactions,^37^ which remain important at larger distances, would be the ones that rule the magnetic behavior of the system. In addition, the surface effects are not negligible due to the lack of translational symmetry, the low coordination number of magnetic ions,^9^ and the existence of broken magnetic exchange bonds, which are responsible for the surface spin disorder.^38^

The irreversibility temperature onset between the experimental ZFC and FC curves is around 295 K that reflects the strong magnetic interaction. The experimental FC curve grows weakly as the temperature decreases and shows a tendency towards saturation below *T*_B_, but also it shows clear differences concerning the theoretical FC curve. This finding corroborates the presence of magnetic interactions^37,38^ in the system.

**Fig. 4:**
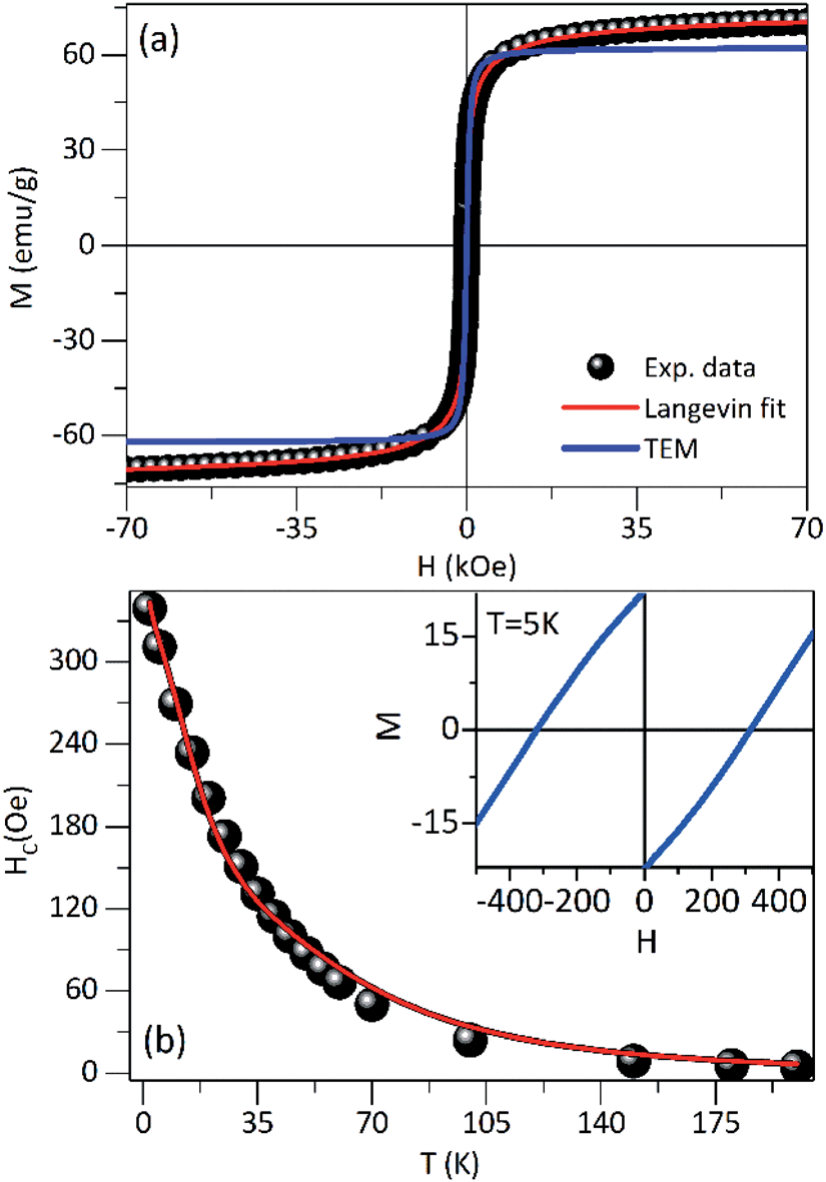
(a) Magnetization *vs.* magnetic field loop of the NPs at 300 K. The fit using the Langevin model (red solid line) is included. The modeling with parameters obtained from TEM data analysis is also included (black solid line). (b) The temperature dependence of the coercive field was fitted with the model proposed. The inset shows the hysteresis loops of the NPs obtained at 5 K.

The hysteresis loops measured at 300 K for PEI-Fe_3_O_4_ NPs are shown in Fig. 4a. The saturation magnetization (*M*_S_) does not reach a saturation value, neither at 300 K nor 5 K, even at the highest applied field of 7 T. Suggesting that the occurrence of a strong anisotropy field and/or magnetic disorder at the surface layer,^39^ that makes difficult the alignment along the field direction. To estimate the *M*_S_, we use the approach to saturation model,^40^ providing *M*_S_ values of 66.5 Am^2^ kg^−1^ and 79.4 Am^2^ kg^−1^ for 300 K and 5 K, respectively. The smaller values in comparison to the one expected for bulk magnetite (*M*_S_ ≈ 92 Am^2^ kg^−1^)^41^ is due to the non-magnetic mass present in our samples. We also observed the absence of coercive field and remanence magnetization above 150 K, confirming the superparamagnetic regime above this temperature, where the thermal energy is predominant and the orientations of magnetic moments are random, so the *M*(*H*) curve shows reversible trend.

The *M*(*H*) curve at 300 K can be fitted to the Langevin function^42^ since relaxed states are expected. Accordingly, the magnetization is described by 
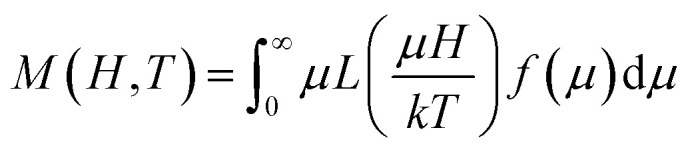
, where the log-normal distribution of magnetic moments is considered, and the mean magnetic moment (〈*μ*〉) of each particle is related to its volume (〈*V*〉) by 〈*μ*〉 = *M*_S_〈*V*〉,^43–45^ where *M*_S_ is the saturation magnetization. The best-fitting is achieved with the parameters *μ*_0_ = 9747*μ*_B_, and *σ* = 1.61 as shown in Fig. 4a, where *μ*_0_ is the median of the distribution related to the mean magnetic moment and *σ* is the polydispersion index. From these results, we can estimate the mean magnetic moment value given by 〈*μ*〉 = *μ*_0_ exp(*σ*^2^/2). Assuming spherical particles and using the equation: 〈*μ*〉 = *M*_S_〈*V*〉 = π*M*_S_〈*D*〉^3^/6, a value of 〈*D*〉 ≈ 10.1 nm, is estimated for the mean particle size. This value is in excellent agreement with the particle size value determined from TEM data analysis (∼9.6 nm). Besides, Fig. 4a shows a simulated *M*(*H*) curve considering parameters obtained from TEM and the Langevin function. This result suggests that the relaxed states (superparamagnetic state of non-interacting particles) is influenced by the magnetic intra- and interparticle interactions and the magnetic polydispersion index.

In Fig. 4b is showing the *H*_C_*vs. T* curve obtained from the *M*(*H*) curves at different temperatures for the PEI-Fe_3_O_4_ NPs. As it is observed, the *H*_C_ shows an increase with temperature decrease. The inset of Fig. 4b shows the *M*(*H*) curve measured at 5 K, with an *H*_C_ ∼ 311 Oe. It is known *H*_C_ is very sensitive to factors such as anisotropy type,^14^ size and distribution of particles, morphology, surface spin disorder, and interparticle interaction.^37^ In our sample, we must consider the surface disorder, and the interparticle interaction which delays the thermal relaxation of the magnetic moments of NPs, are predominant factors that determine the value of *H*_C_.

However, the temperature dependence of the coercive field can be modeled taking into account the particle size distribution (distribution of *T*_B_ obtained from the ZFC–FC analysis) and/or the interaction effects. The coercive field is given by 〈*H*_C_〉_*T*_ = *M*_r_(*T*)/(*χ*_r_ + *M*_r_(*T*)/*H*_CB_(*T*)), where *M*_r_ is the remanence magnetization, *χ*_r_ is the susceptibility of the superparamagnetic particles, and *H*_CB_ is the coercive field of blocked particles. An empirical parameter *γ* is included in *f*(*γT*_B_) which is related to the particle interactions.^34,46^ As shown in the main panel of Fig. 4b, the fit provides a *γ* = 0.8, which is close to the value expected for systems with negligible interparticle interactions (*γ* = 1).^46^

**Fig. 5:**
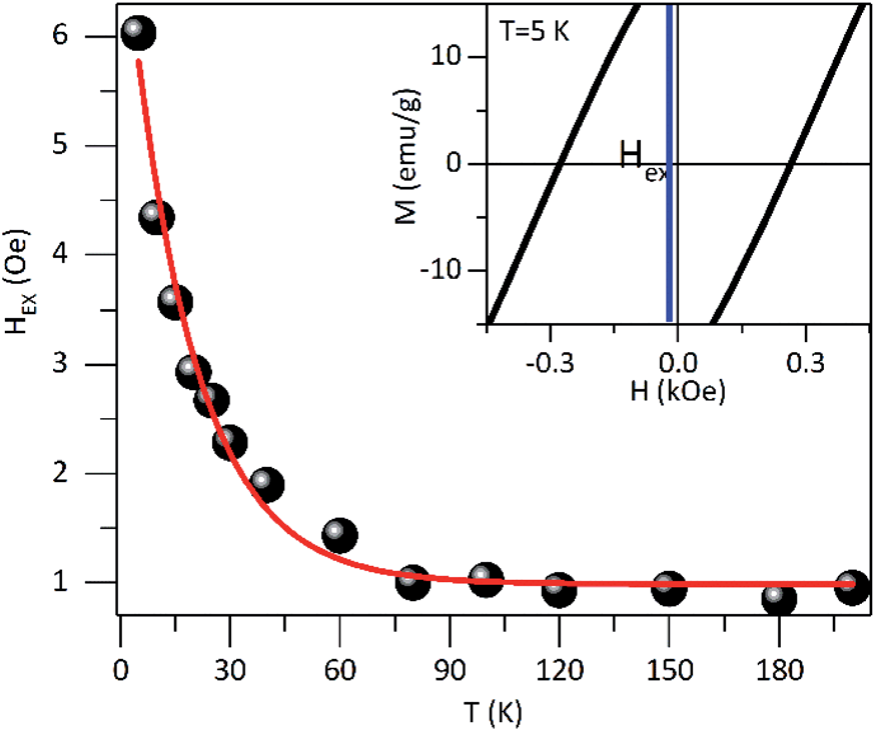
Exchange bias *H*_EX_ anisotropy field as a function of the temperature obtained after field cooling the samples with a field of *H*_FC_ = 1591.5 kA m^−1^. The inset shows the shift of the hysteresis loop at 5 K, which defines the *H*_EX_ for PEI-Fe_3_O_4_ NPs.

In order to explore possible exchange coupling effects between the disordered surface spins and the magnetic core, we measured the *M*(*H*) loops at low temperature after field-cooling the sample with a field *H*_FC_ = 1591.5 kA m^−1^, to ascertain the presence of an EB anisotropy field (*H*_EX_). Fig. 5 shows the presence of an exchange field^47^ for temperatures below ≈60 K, with a thermal dependence of exponential type down to *T* = 5 K.

The *H*_EX_ origin was assigned to the coupling of a layer of disordered spins at the particle surface and a well-ordered region of spin in the core region of the particle. The core spins exert torque on the surface spins that do not follow the anisotropy direction of the core due to the disorder, leading to the *H*_EX_ occurrence. To evaluate the exchange anisotropy at low temperatures we use the *H*_EX_ thermal dependence, given by *H*_EX_(*T*) = *H*_EX_(0)exp(−*BT*) where *H*_EX_(0) is the EB field at *T* = 0 K and *B* is a constant. The fit provides a *H*_EX_(0) = 0.5 kA m^−1^ and *B* = 5.5 10^−2^ K^−1^. The small value of *H*_EX_(0) shows the EB effect is rather weak, and it seems to be associated with the magnetic coupling between the disordered surface spins and the magnetic core. This is consistent with previous reports on core–shell Au/Fe_3_O_4_ NPs where the disordered spins are present at both the inner and outer surface of the magnetite shell in the Au/Fe_3_O_4_ NPs.^34^

The *H*_EX_ low value indicates that the two magnetic regions are weakly coupling, where the effective anisotropy of the core is expected to be greater to achieve the reversal of the surface spins region and where the surface spins rearrange faster than core spins. It is worth mentioning that we are considering just the intrinsic origin of *H*_EX_ and dipolar interactions do not affect the value of *H*_EX_, which could result in *H*_EX_ reduction in large cooling fields^18^ and *H*_EX_ increase in small particles due to the fact that shell is thicker than core.^17^

**Fig. 6:**
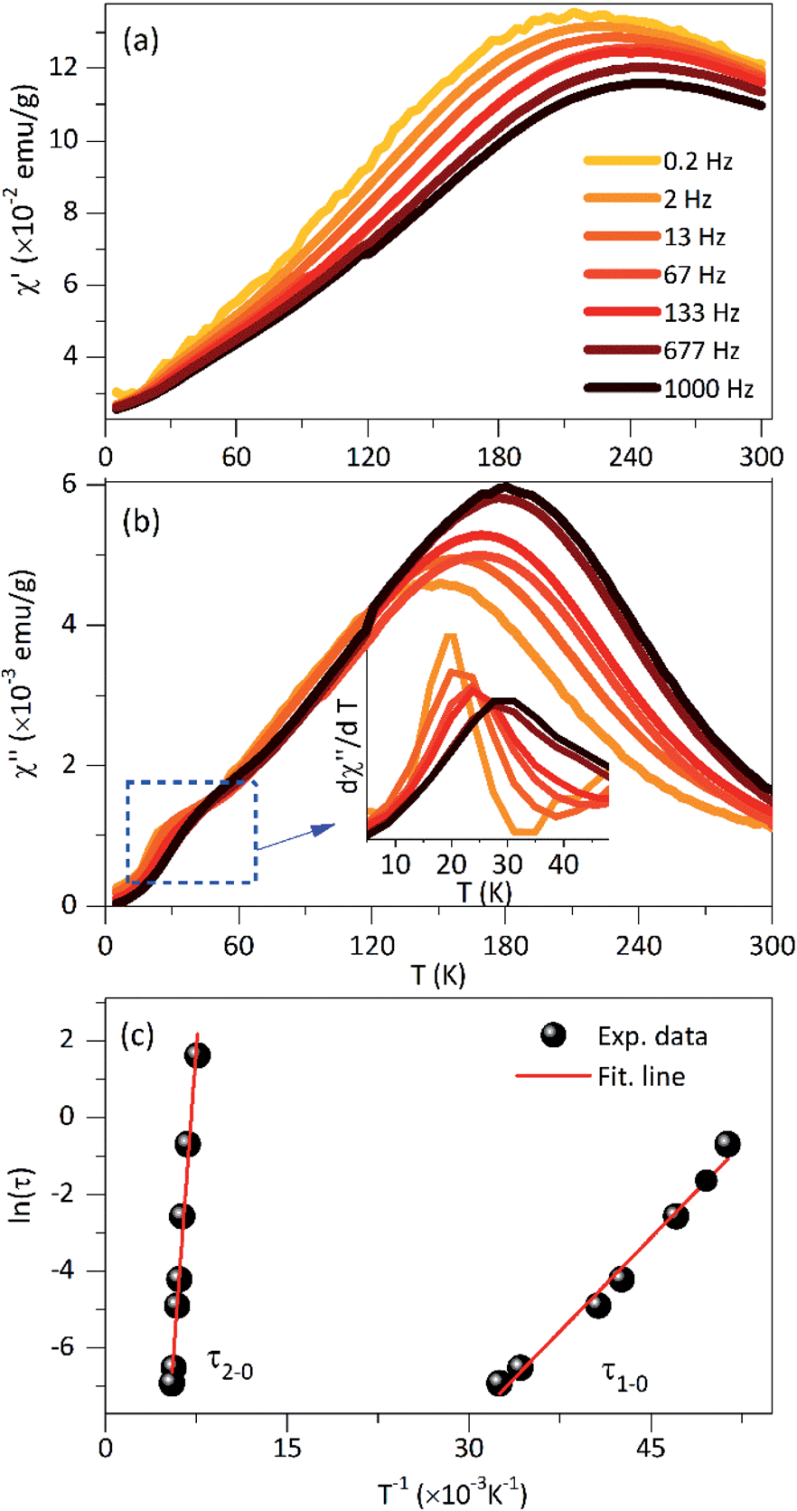
AC-magnetic susceptibility, (a) in-phase component *χ*′(*T*), at different excitation frequencies, and with an oscillating field of 0.4 kA m^−1^ for PEI-Fe_3_O_4_ NPs. (b) The out-of-phase component *χ*′′(*T*) curve and the inserted d*χ*′′/*T* × d*T* curve are shown. (c) Analysis of the relaxation time as a function of the inverse of *T*_m_ using the Néel–Arrhenius relation obtained from the imaginary component *χ*′′(*T*).

Fig. 6 shows the in-phase (*χ*′) and the out-of-phase (*χ*′′) susceptibility components as a function of temperature with a range from 0.2 Hz to 1 kHz and in an oscillating magnetic field of 0.08 kA m^−1^. As was observed, the peak position of *χ*' × *T* curve is located at ∼200 K for the lowest frequency (0.2 Hz) and this maximum was shifted to higher temperatures with the frequency increase. This high-temperature peak is expected for NPs with interparticle interactions and this behavior was also observed in *χ*′′ × *T* curve (Fig. 6b). Also, a shoulder in the low temperature that clearly shows a dependence on frequency is evidenced, as shown in the d*χ*′′/*T* × d*T* curve (inset on Fig. 6b). It could be attributed to non-interacting particles. Other low-temperature reports were assigned to a significant amount of surface spin disorder at 15 K for 7 nm maghemite NPs^48^ while spin-glass-like-transition at 35 K for 40 nm magnetite NPs.^19^

Considering that non-interacting particles where the barrier energy is determined by the uniaxial anisotropy, the spin relaxation is a thermally activated process, as is proposed by the Néel–Arrhenius model, *τ* = *τ*_0_ exp(*E*_a_/*k*_B_*T*), where *E*_a_/*k*_B_ is the activation energy. Assuming that the temperatures in *χ*′′(*T*,*f*) correspond to the *T*_B_ and the low-temperature peak is best observed in d*χ*′′/d*T vs. T* curve (inset on see Fig. 6b). The ln(*τ*) × 1/*T* curve fit (see Fig. 6c) provides values of *τ*_1–0_ = 1.9 10^−8^ s, *E*_a_/*k*_B_ = 325 K and *τ*_2–0_ = 9.8 10^−14^ s, *E*_a_/*k*_B_ = 4224 K for the low and high temperature peaks, respectively. The characteristic time *τ*_1–0_ is very close to the values reported for non-interacting NPs in an applied field (10^−9^–10^−11^ s) close to zero, while the *τ*_2–0_ is consistent with interacting NPs.^39^

The high-temperature peak analysis with the Neél model suggests taking into account the presence of interparticle interactions, and that can be assessed using the Vogel–Fulcher model,^49^*τ* = *τ*_0_ exp(*E*_a_/(*k*_B_(*T*_max_ − *T*_0_))), where *T*_0_ is a characteristic temperature which magnifies the interaction energy among the NPs and *T*_max_ is the onset temperature of the blocked state. Considering a characteristic time of *τ*_0_ = 10^−9^ s, he obtained parameters from the experimental data fit are *T*_0_ ∼ 56 K and *E*_a_/*k*_B_ ∼ 1760 K. According to what was reported by Yasin *et al.*, when *T*_0_ > 0 has a collection of interacting spins; meanwhile, when *T*_0_ < 0 indicates a spin-glass system.^50^

Besides that, in the absence of an applied low magnetic field, *E*_a_ is given by *E*_a_ = *K*_eff_*V*, where *K*_eff_ is the effective magnetic anisotropy constant. Using the NPs size obtained from TEM data analysis, we estimated a *K*_1-eff_ = 10^4^ J m^−3^ for the low-temperature peak. The *K*_1-eff_ value is very close to the magnetocrystalline value of bulk magnetite (1.35 10^4^ J m^−3^).^51^ In addition, the value obtained from *E*_a_/*k*_B_ was used to estimate the NPs mean diameter (considering them as spherical). Using *K*_eff_ of bulk magnetite, a diameter size of ∼8.7 nm was calculated being this very close to the size found by TEM while ∼21 nm corroborates the presence of agglomerated NPs.

For further characterization, we used the empirical parameter, *ϕ*, given by:^49^*ϕ* = Δ*T*_f_/*T*_f_Δlog_10_(*f*), which is the relative shift of the peak temperature (*T*_f_) per AC frequency decade change. The experimental data provides a value of *ϕ* = 0.14 for the low-temperature peak. This value is very close to the ones reported for non-interacting particles or superparamagnetic systems (0.10 < *ϕ*< 0.13). For the high-temperature peak, a *ϕ* = 0.071 was determined, which is in the range of values corresponding to interacting particles (0.03 < *ϕ* < 0.1).^51^ From these results, we can infer that the studied system contains both non-interacting NPs that present superparamagnetic behavior and interacting NPs associate with the aggregates that show collective response even to the presence of PEI.

### 3.3 Power absorption

**Fig. 7:**
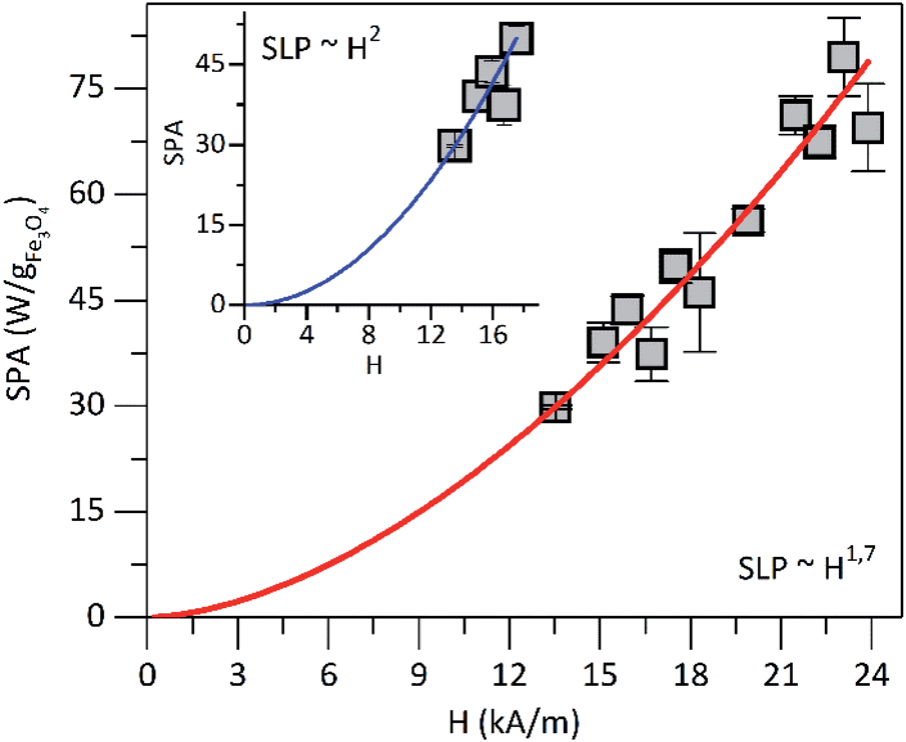
SLP *vs.* magnetic field amplitude at 571 kHz. The line presents the best fit using SLP ≈ *H*^*λ*^. In the inset is shown the SLP *vs.* magnetic field amplitude in the low magnetic field until 17.51 kA m^−1^.

Further characterization was performed to assess the heating ability of PEI-FE_3_O_4_ NPs in the aqueous medium at pH 7. In order to determine the magnetic properties impact (as surface effects, interparticle interactions) in the heating efficiency of the superparamagnetic PEI-Fe_3_O_4_ NPs which is quantified by in the specific loss power (SLP). In Fig. 7 is shown the SLP value which increases with the increasing field in agreement with reports in the literature.^52^

For fields below 17.51 kA m^−1^ and using the power law, the SLP displays a field dependence of SLP ∼ *H*^2^ (see the inset in Fig. 7), which is in agreement with the linear response theory (LRT) for superparamagnetic particles as predicted by Roseinsweig.^53^ However, for all measured field values, the dependence is SLP ∼ *H*^1.7^, which is not predicted by the LRT. On the other hand, the measured SLP value at 23.87 kA m^−1^ was of ∼70 W g^−1^ for the PEI-Fe_3_O_4_ NPs studied in this work. In Table 1 is summarized the SLP values reported in the literature for iron oxide NPs with different sizes, surface coatings, AMF, and frequency. The SLP value obtained in this work is comparable to the values reported in the literature as observed in Table 1. Therefore, we concluded that the parameters followed in this work were optimized in such a way that the efficiency to transform electromagnetic energy into heat was higher than those reported in the literature. Furthermore, the differences among the SLP values seems to be related to the dependence on parameters such as particle size distributions,^54^ coating, NPs aggregation, hydrodynamic size, presence of magnetic interactions,^55^ magnetic anisotropy,^56^ magnetic volume,^57^ and/or different experimental conditions, among others. All these factors can affect the LRT behavior of power loss efficiency.

**Table 1:** SLP values of magnetic NPs reported in the literature with approximate sizes and different surfaces coatings

Name	Surface coating	Core size [nm]	SLP [W g^−1^]	Measured conditions [kA m^−1^]/[kHz]	Ref.
Fe_3_O_4_	PEI-poly(acrylic acid-*co*-maleic acid)	10.3	15	7.28/621.7	58
Fe_3_O_4_	Polyethylene glycol	11.0	4.5	3.2/600	59
Fe_3_O_4_		10.0	16	∼8/524.2	60
Iron oxide^a^	Oleic acid-oleylamine-trioctylphosphine oxide	8.8	80.7	23.87/580.5	61
Fe_3_O_4_		10.2	63	9.8/276	62
Fe_3_O_4_	PEI-poly(acrylic acid-*co*-maleic acid)	10.0	12.15	11.94/111	63
Fe_3_O_4_	Oleic acid	8.0	33.5	26.6/265	64
Fe_3_O_4_	PEI	9.6	70	23.87/571	This work

a The solvent used was hexane and water, in the others.

## 4. Conclusions

PEI-coated Fe_3_O_4_ NPs of narrow size distribution and mean size of ∼10 nm were successfully synthesized by a one-step co-precipitation route. The systematic investigation of the magnetic properties suggests the presence of non-interacting particles showing a superparamagnetic behavior with a low blocking temperature (∼35 K) and interacting particles, likely forming agglomerates, with a higher blocking temperature (>150 K), in which the surface spin disorder is weak and dominated by interparticle interactions. A high SLP value of ∼70 W g^−1^ was obtained (at 571 kHz and 45 kA m^−1^). Therefore, the magnetic properties play a crucial role in determining the heating efficiency obtained in this work, which is attractive for future application.

## Conflicts of interest

The authors declare no competing financial interest.

## Supplementary Material
